# High temperature anomalous Raman and photoluminescence response of molybdenum disulfide with sulfur vacancies

**DOI:** 10.1038/s41598-023-43756-w

**Published:** 2023-09-29

**Authors:** M. K. Ranjuna, Jayakumar Balakrishnan

**Affiliations:** Department of Physics, Indian Institute of Technology Palakkad, Palakkad, Kerala 678623 India

**Keywords:** Materials for devices, Materials for optics, Materials science, Two-dimensional materials

## Abstract

We report an intriguing anomalous behavior observed in the temperature-dependent Raman spectra of mono-, bi-, and trilayer molybdenum disulfide samples with sulfur vacancies, measured at high temperatures ranging from room temperature to 463 K. In contrast to existing reports, we observed a decrease in the FWHM of the A$_{1g}$ phonon mode, along with an increase in the relative intensity of the A$_{1g}$ mode to the E$_{2g}^1$ mode, as the temperature increased. This trend becomes less prominent as the layer number increases from monolayer, disappearing entirely in few-layer samples. Additionally, we observed an intensity enhancement in the photoluminescence spectra of MoS_2_ samples at high temperatures (up to 550 K), which depends on the layer number. These observations are explained by considering the presence of sulfur vacancies, their interaction with the environment, electron density reduction, and a phonon-mediated intervalley charge transfer at elevated temperatures. Our results unambiguously establish that the effect of defects (sulfur vacancies) is more prominently reflected in the temperature dependence of FWHM and the relative intensity of the Raman modes rather than in the Raman peak positions.

## Introduction

Two-dimensional (2D) crystals of Transition metal dichalcogenides (TMDCs) are emerging materials with properties ranging from semiconducting to metallic to even superconducting. TMDCs are MX_2_-type compounds where M is a transition metal element from groups IV, V, and VI of the periodic table and X is a chalcogen species—S, Se, and Te^1^. Among various TMDCs, molybdenum disulfide (MoS_2_) is a well-studied crystal in the 2D limit. The presence of a direct bandgap in the monolayer limit^2^, remarkable light-matter interactions^3^, strong spin-orbit and Coulomb interactions^4^, efficient valley-selectivity^5^, the existence of strongly correlated many-body quasi-particles like trions at room temperature^6,7^ as well as superconductivity^8,9^, makes this emerging material interesting for fundamental studies as well as for novel technological applications.

Optical measurement techniques like Raman and photoluminescence (PL) spectroscopy are considered versatile tools for understanding various properties of MoS_2_ and other TMDC 2D crystals. In general, the optical properties of TMDC are strongly influenced by the material parameters like layer number^10^, stacking order^11^ and defects^12,13^, as well as external factors like the presence and choice of substrate^14^, temperature^15^, strain^16^, doping level^17^ and applied magnetic field^18^. A systematic analysis of various optical properties can provide information about layer number, stacking order, defect states, interactions between various carriers, energy transfer processes, and splitting and evolution of energy bands in the crystal. Moreover the peak position, full width at half maximum (FWHM) and relative intensity/integrated intensity of Raman spectrum measured at different temperatures can provide additional information like anharmonicity in lattice potential^19–21^, thermal expansion^22,23^, phase transitions^24^ and thermal conductivity of the crystal^21,25^.

Even though there are reports on the evolution of Raman and PL spectra of MoS_2_ 2D crystals with temperature, a comprehensive report which accounts for the layer number and stacking order variations is still lacking. Hence, in the present work, we carried out temperature-dependent Raman and PL measurements of mechanically exfoliated mono-, bi-, tri-, and few-layer MoS_2_ crystals on Si/SiO_2_ substrate, including the 2H and 3R stacked bilayers. Our results clearly demonstrate an anomalous behaviour in the Raman active A$_{1g}$ mode. The FWHM of the A$_{1g}$ mode decreases as the temperature is increased while the relative intensity of A$_{1g}$ mode to E$_{2g}^1$ mode shows an increase with the increase in temperature. Interestingly this trend is significant in monolayer samples and decreases as the layer number increases and becomes less significant in few-layer flakes. The 2H and 3R stacked samples show similar responses. To understand the underlying mechanism, we performed additional photoluminescence measurements, where a layer number-dependent intensity enhancement in the PL spectra of MoS_2_ crystals at high temperatures is observed. These observations are explained by considering (I) the sulfur vacancies in MoS_2_ samples, (II) the interaction of sulfur vacancies with the environment and electron density reduction at higher temperatures, and (III) the intervalley charge transfer of thermally generated carriers.

## Experimental mthods

### Sample preparation and characterization

The MoS_2_ flakes are mechanically exfoliated from bulk crystals using scotch tape and transferred to a heavily p-doped 500 $\mu$m thick Si wafer with a 285 ± 5 nm thermally oxidized layer (Si/SiO_2_). The layer number of the flakes is initially identified from their optical contrast using a microscope with white light illumination. The layer number and stacking order of the flakes are further confirmed by their Raman spectra captured with 532 nm laser excitation. The relative separation between the position of high-frequency Raman modes (E$_{2g}^1$ and A$_{1g}$) and the position of low-frequency shear mode are used to determine the layer number of the flakes. The stacking order of exfoliated bi- and trilayers are determined from the low-frequency Raman modes. In all cases, measured spectra are fitted with Lorentzian function to estimate the spectral parameters: peak position, FWHM, intensity (height) and integrated intensity (area).

### Raman and photoluminescence spectroscopy

Raman and PL measurements are performed in a HORIBA LabRAM HR Evolution Raman spectrophotometer setup using an Nd-YAG laser of wavelength 532 nm in the off-resonance excitation. For all temperature-dependent measurements, excitation laser power is kept at  200 μW to avoid local heating. The flakes are focused using a $\times$ 50, NA = 0.5 objective of spot size $< 0.55 \mu$m radius. For Raman and PL spectra, we use gratings with 1800 lines per mm and 600 lines per mm, respectively. To perform measurements at elevated temperatures and in air and N_2_ environment, a Linkam HFS600E-PB4 temperature-controller stage is used. Raman and PL spectra measurements are carried out on the exfoliated samples before and after annealing. Annealing is performed in a flow tube furnace, where the samples are kept at 623 K for 3 hours in a Hydrogen (5%) in Argon (95%) environment. Raman and PL measurements are performed over the spectral range of 10–600 cm$^{-1}$ and 1.4–2.29 eV, respectively. Laser power-dependent measurements are carried out at ambient conditions using a step-variable neutral density (ND) filter.

## Results and discussion

**Figure 1:**
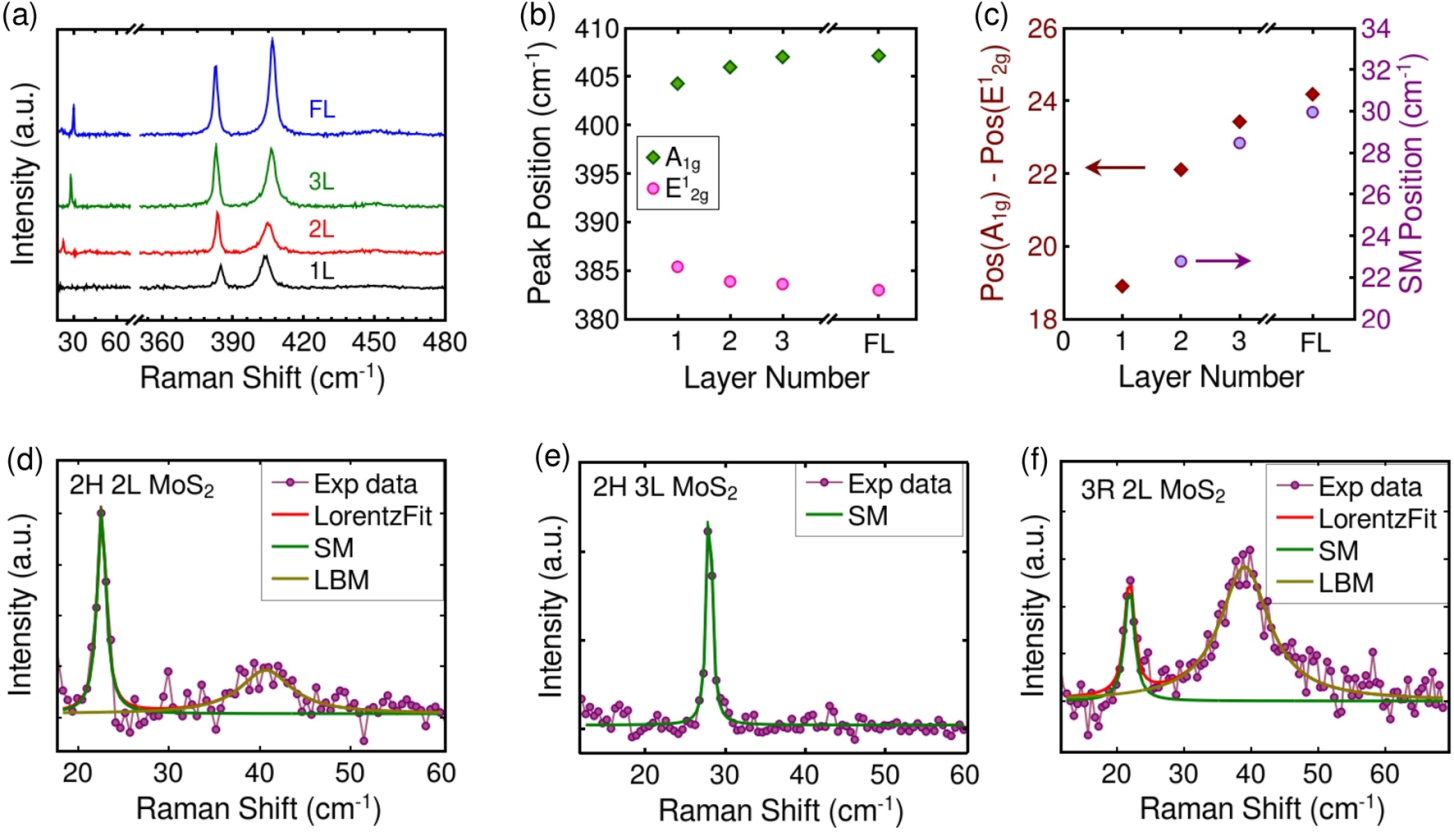
(**a**) Representative Raman spectra of mono-, bi-, tri- and few-layer MoS_2_ (**b**) Variation of E$_{2g}^1$ and A$_{1g}$ phonon mode frequencies with layer number (**c**) The relative separation between E$_{2g}^1$ and A$_{1g}$ phonon modes (rhombus) and the frequency of shear mode (circle) as a function of layer number of MoS_2_. Low frequency Raman modes of (**d**) 2H stacked bilayer, (**e**) 2H stacked trilayer and (f) 3R stacked bilayer MoS_2_ crystals.

Figure 1a illustrates the evolution of Raman spectra with layer number of MoS_2_ crystals exfoliated on Si/SiO_2_ substrate. The high-frequency Raman peaks around 385 cm$^{-1}$ and 405 cm$^{-1}$ are labelled as E$_{2g}^1$ and A$_{1g}$, respectively. The E$_{2g}^1$ mode originates from the doubly degenerate in-plane vibrations of the molybdenum (Mo) and sulfur (S) atoms. The A$_{1g}$ mode originates from the out-of-plane vibrations of S atoms^26^. Figure 1b shows the frequency of the E$_{2g}^1$ and A$_{1g}$ as a function of layer number. As the layer number increases, the peak position of E$_{2g}^1$ mode (Pos E$_{2g}^1$) decreases, and that of A$_{1g}$ (Pos A$_{1g}$) mode increases i.e. the relative separation between the intralayer modes increases as the layer number increases (see Fig. 1c). In addition, the Raman active inter-layer shear mode (SM) and layer breathing mode (LBM) also show layer number dependence in MoS_2_^27^. Shear mode and layer breathing mode originate from in-plane and out-of-plane vibrations of both S and Mo atoms. These modes are absent in the monolayer, and the frequency of the shear mode increases as the layer number increases from bilayer to bulk; see Fig. 1c. The stacking order of bilayer and trilayer flakes are identified from their low-frequency Raman spectra; see Fig. 1d–f. The integrated intensity ratio of LBM to SM distinguishes the stacking order in bilayer crystals^28^. In the case of trilayer samples with 2H stacking, both the layer breathing and shear mode appear $\sim$28 cm$^{-1}$ and emerges as a single peak. Hence, identifying a 2H stacked trilayer is much easier than other trilayer polytypes. Above four-layer, accurately determining layer number and stacking order from the Raman spectrum is difficult due to less differences in spectral parameters and the possibility of different polytypes. Hence, in the present work, any thin samples with a layer number higher than four are labelled as few-layer without assigning any stacking order. Refer the section S1 of the electronic supporting information file for further details on the layer number and stacking order characterization.

### Temperature dependent Raman measurements

#### Uniform heating

**Figure 2:**
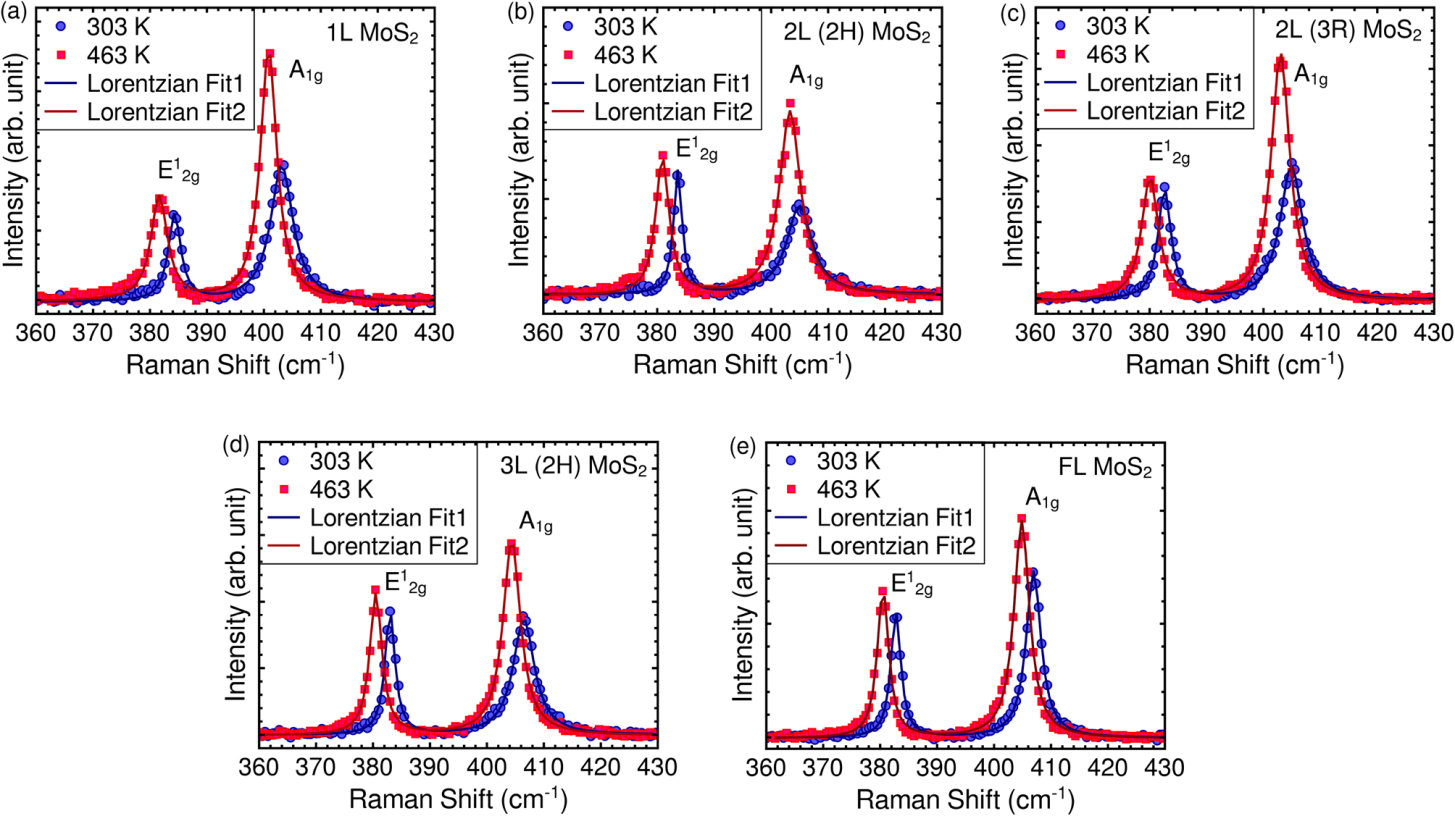
Comparison of Raman spectra acquired at the lowest and highest temperatures under study for (**a**) monolayer, (**b**) 2H and (**c**) 3R stacked bilayers (**d**) 2H stacked trilayer and (**e**) few-layer MoS$_{2}$ crystals on Si/SiO_2_ substrate.

After characterizing the MoS_2_ samples in terms of layer numbers and stacking order, temperature-dependent Raman measurements are conducted under ambient conditions. The measurements spanned a temperature range from room temperature up to 463 K. The acquired Raman spectra at different temperatures are presented in Figure S3 of the supporting information file. To aid in visualizing the impact of temperature, a comparison of the Raman spectra obtained at the lowest and highest measured temperatures is shown in Fig. 2. As the sample temperature increases, there is a significant enhancement in the intensity of both the E$_{2g}^1$ and A$_{1g}$ modes across all layer numbers and stacking orders. Additionally, there is a redshift observed in the frequencies of these Raman modes. The variations of each spectral parameter with temperature are analyzed individually in the subsequent sections.

**Figure 3:**
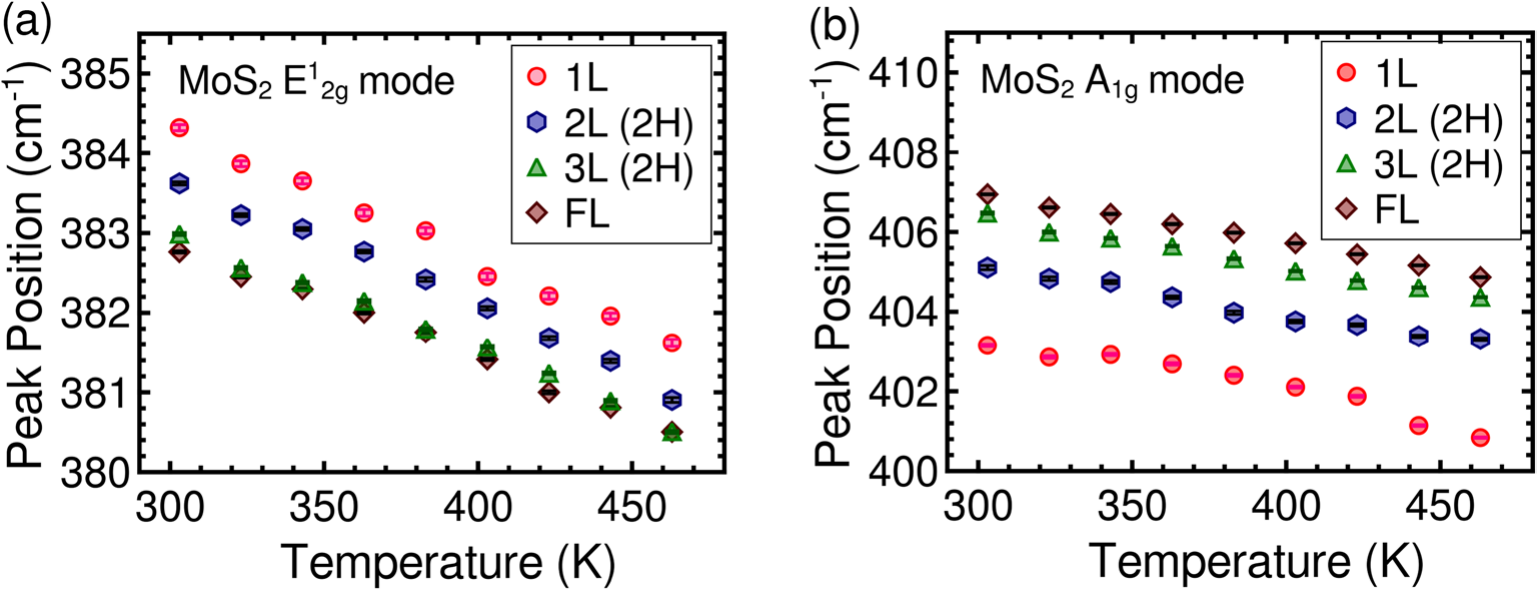
The variation of (**a**) E$_{2g}^1$ and (**b**) A$_{1g}$ peak position of MoS_2_ crystals on Si/SiO_2_ substrate as a function of measured temperature.

**Figure 4:**
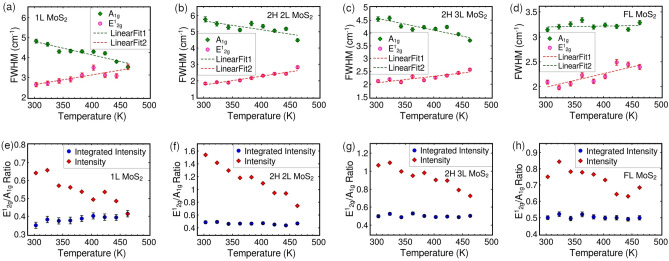
The variation of FWHM of E$^1_{2g}$ and A$_{1g}$ Raman modes in (**a**) monolayer, (**b**) 2H-stacked bilayer, (**c**) 2H-stacked trilayer and (**d**) fewlayer MoS_2_ crystals on Si/SiO_2_ substrate as a function of measured temperature. The dashed green and pink lines are given for better visualization of the trend. The variation of intensity ratio and integrated intensity ratio of E$^1_{2g}$ mode to A$_{1g}$ mode in (**e**) mono-, (**f**) 2H-stacked bi-, (**g**) 2H-stacked tri- and (**h**) few-layer MoS_2_ crystals on Si/SiO$_2$ substrate as a function of measured temperature.

The variation in peak position of the high-frequency Raman modes is presented in Fig. 3. As the sample temperature increases, both phonon modes exhibit a linear redshift in their frequencies, consistent with previous reports that attribute this temperature dependence to lattice thermal expansion and thermal anharmonicity^19,21,28^. The data is fitted with a linear function to obtain first-order temperature coefficients of each sample and the obtained values demonstrate good agreement with previous reports involving MoS_2_ on Si/SiO_2_ substrates^19,28,29^, see Table S2. The variation of FWHM of the E$_{2g}^1$ and A$_{1g}$ modes with temperature is shown in Fig. 4. At room temperature, the A$_{1g}$ mode displays a higher FWHM compared to the E$_{2g}^1$ mode across all layer numbers and stacking orders. This higher FWHM of A$_{1g}$ is in agreement with the reduction in its phonon lifetime due to the combined effect of phonon-phonon and phonon-electron interactions^19^. Previous reports show that the FWHM of both E$_{2g}^1$ and A$_{1g}$ modes remains independent of temperature below 100K and increases linearly as the temperature increases further^19,21^. However, in the present work, we observed that as the temperature increases, the FWHM of the E$_{2g}^1$ mode increases, while that of the A$_{1g}$ mode decreases in mono-, bi-, and tri-layer samples. Moreover, with an increasing layer number, the temperature-induced reduction in FWHM of A$_{1g}$ mode diminishes and disappears when the sample thickness reaches a few layers. This newly observed temperature-induced response of the A$_{1g}$ mode requires further investigations to understand its underlying mechanism and is discussed in the following sessions.

Additionally, we analyzed the variation in the relative intensity of high-frequency modes with temperature. The relative intensity of the E$_{2g}^1$ mode to the A$_{1g}$ mode decreases as the temperature increases, while the integrated intensity ratio remains constant, as depicted in Fig. 4. However, with uniform heating, as the temperature increases, the intensity of both the E$_{2g}^1$ and A$_{1g}$ modes increases, as shown in Fig. 2. The overall outcome of uniform heating is the temperature-induced sharpening of A$_{1g}$ mode, in contrast to the temperature-induced broadening observed in the E$_{2g}^1$ mode. To confirm the reliability and repeatability of this phenomenon, we performed measurements on different samples before and after annealing; refer to Table 1 and figures in Section S3 for details. Both the 2H and 3R stacked MoS$_2$ samples exhibited similar trends, as illustrated in Figure S6 of the supporting information file.

**Table 1:** Summary of MoS$_2$ samples used for Raman measurements.

Layer number	Number of samples used
1L	3
2L (2H)	3
2L (3R)	1
3L (2H)	4
FL	2

**Figure 5:**
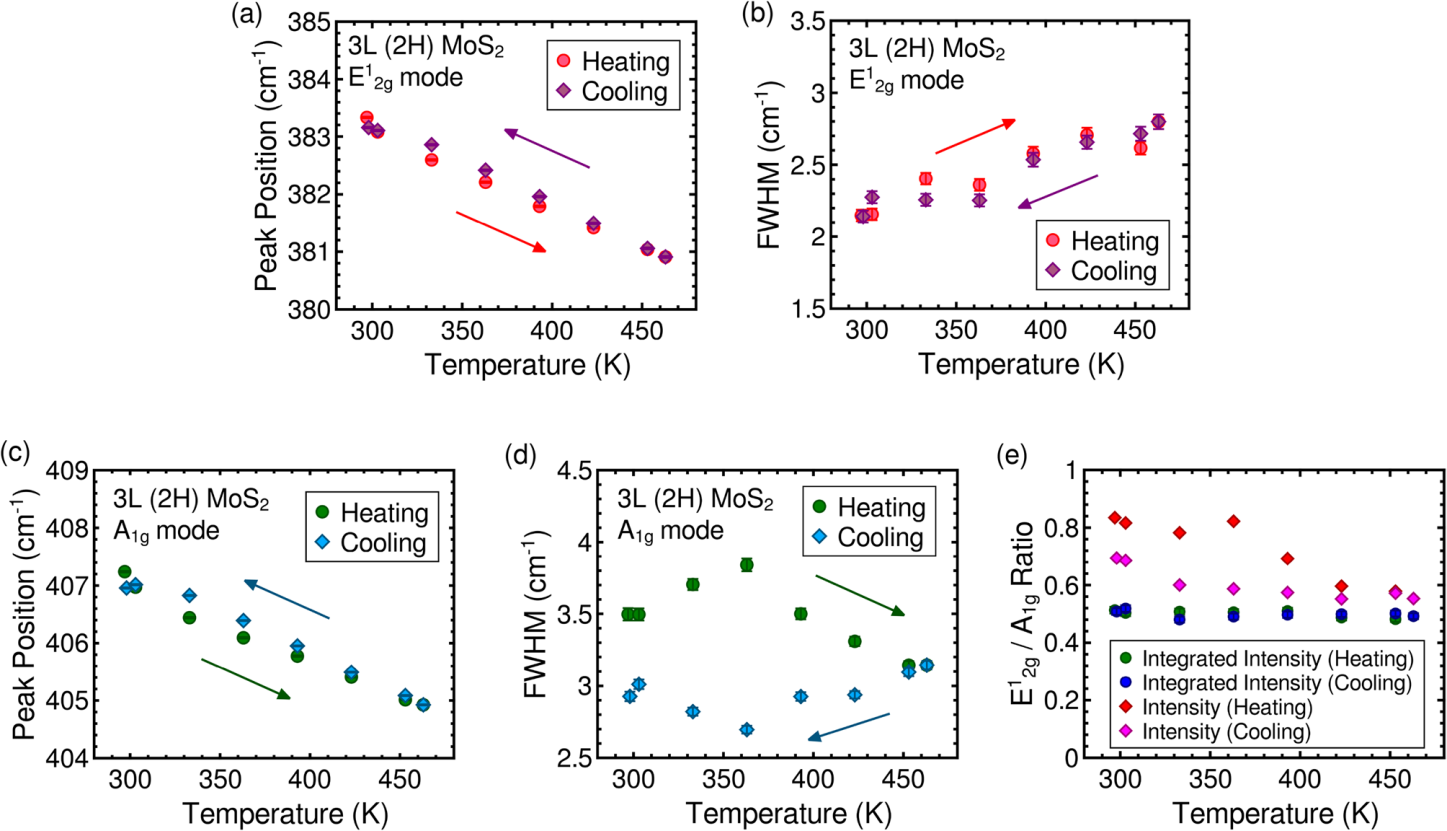
Variation of (**a**) E$^1_{2g}$ peak position, (**b**) FWHM, (**c**) A$_{1g}$ peak position, (**d**) FWHM and (**e**) E$^1_{2g}$ to A$_{1g}$ intensity ratio and integrated intensity ratio of 2H stacked trilayer MoS$_2$.

Furthermore, temperature-dependent Raman measurements are conducted by raising and lowering the sample temperature, and the findings for a representative 2H stacked tri-layer sample are summarized in Fig. 5. The peak position and FWHM of the E$_{2g}^1$ mode exhibit consistent behavior in both heating and cooling cycles. However, in the case of the A$_{1g}$ mode, while the peak position shows a similar response in both heating and cooling cycles, the FWHM displays clear hysteresis. The relative intensity ratio of the E$_{2g}^1$ mode to the A$_{1g}$ mode also exhibits hysteresis, whereas hysteresis is absent in the integrated intensity ratio. This hysteresis behavior is exclusively observed in the anomalous responses of the A$_{1g}$ mode. The emergence of this hysteresis suggests that these anomalous responses are associated with interactions or phenomena occurring at the surfaces at higher temperatures.

#### Local laser heating

**Figure 6:**
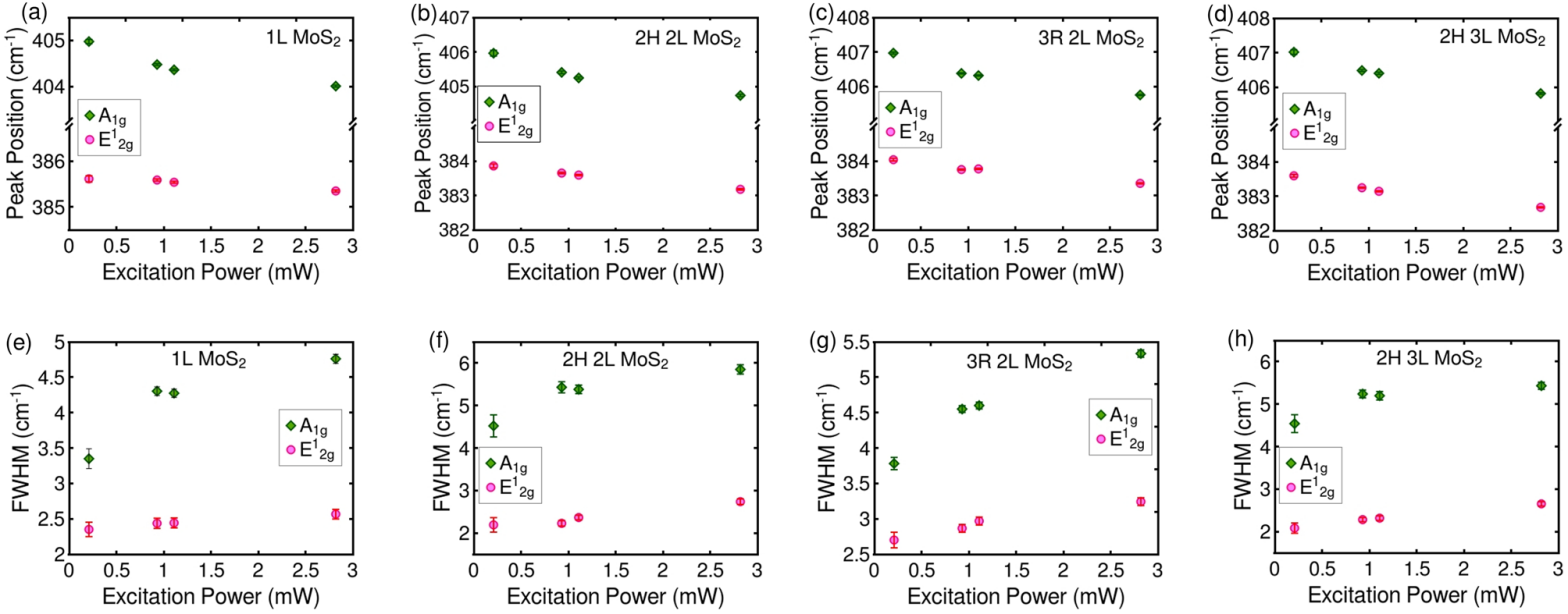
Variation of peak position of high frequency Raman modes with excitation laser power for (**a**) monolayer, (**b**) 2H and (**c**) 3R stacked bilayers and (**d**) 2H stacked trilayer MoS$_{2}$ crystals on Si/SiO$_2$ substrate. Variation of FWHM of E$_{2g}^1$ and A$_{1g}$ modes with excitation laser power for (**e**) monolayer, (**f**) 2H and (**g**) 3R stacked bilayers and (**h**) 2H stacked trilayer MoS$_{2}$ crystals on Si/SiO$_2$ substrate.

In addition to temperature-dependent Raman measurements using uniform heating, we also performed Raman measurements with localized laser heating. By employing higher laser powers, one can achieve local heating of the sample, resulting in a Gaussian temperature profile within the 2D material. Figure 6 illustrates the variation of peak position and FWHM of the high-frequency phonon modes with excitation power. As the laser power increases, the phonon frequency of both the E$_{2g}^1$ and A$_{1g}$ modes decreases. However, the FWHM of both phonon modes increases with higher excitation power across all samples. These observations are consistent with the previously reported local heating-induced redshift and broadening of Raman modes in MoS$_2$ crystals^25,29–31^. The variation in relative intensity and integrated intensity is provided in Figure S7 of the supporting information file.

Remarkably, the temperature-induced anomalous trend observed in the FWHM of the A$_{1g}$ mode is specific to uniform heating and not observed with laser-induced heating. This disparity highlights the need to explore plausible temperature- and laser power-induced processes that could influence the Raman spectrum of MoS$_{2}$ crystals. Since temperature-induced strain and doping are previously reported in graphene^32,33^, similar phenomena are also expected to occur in other 2D materials. Moreover, during laser power-dependent Raman measurements of supported 2D materials, there arises the possibility for substrate-induced doping^34,35^. This effect is prominent at lower incident powers and reaches saturation at higher laser powers. Concurrently, the influence of local heating becomes more prominent at higher excitation powers. The degree and nature of doping is also influenced by the substrate’s characteristics. Consequently, to elucidate the underlying reasons behind the anomalous behavior of the A$_{1g}$ mode, it is crucial to investigate the plausibility of temperature- and laser power- induced doping and strain, as well as their potential effects on the Raman spectrum of 2D MoS$_{2}$ crystals.

The impact of strain on the Raman spectra of MoS$_{2}$ depends upon the nature of strain developed in the crystal. When a monolayer or bilayer MoS$_{2}$ flake is subjected to mechanical uniaxial tensile strain, the doubly degenerate in-plane E$_{2g}^1$ mode exhibits a redshift. When the increase in strain becomes sufficient to distort the lattice symmetry, it splits into two modes ( E$_{2g}^{1+}$ and E$_{2g}^{1-}$)^36,37^. In contrast, the increased in-plane uniaxial strain has a negligible effect on the frequency of the out-of-plane A$_{1g}$ mode^36–38^. Applying considerable localized strain on atomically thin MoS$_{2}$ results in an observable redshift in the phonon frequency and broadens the E$_{2g}^1$ and the A$_{1g}$ modes^39^. However, the frequency of the A$_{1g}$ mode is less affected than that of the E$_{2g}^1$ mode. For thicker MoS$_{2}$ crystals, the combined effect of both in-plane and out-of-plane uniaxial strain is the blueshift in A$_{1g}$ and E$_{2g}^1$ mode frequencies^16^. In temperature-dependent Raman measurements with uniform heating, the possibility of strain comes from the thermal expansion mismatch of the MoS$_{2}$ crystal and the SiO$_2$ in the substrate. Consequently, a uniform in-plane strain is expected, to which the A$_{1g}$ mode is less sensitive. During excitation power-dependent Raman measurements, local strain may occur due to localized heating and temperature gradients within the sample. In such cases, broadening is anticipated in both the A$_{1g}$ and E$_{2g}^1$ modes.

The nature of doping plays a vital role in the doping-induced Raman response of MoS$_{2}$ crystals. An in situ carrier-dependent Raman study of a top-gated single-layer MoS$_{2}$ transistor demonstrated that the A$_{1g}$ phonon mode frequency redshifts, and the FWHM increases with electron doping^40^. In addition, p-doping in the MoS$_{2}$ is accompanied by a blueshift in phonon frequency and an enhancement in the intensity of the A$_{1g}$ peak^41^. However, in all these experiments, the in-plane E$_{2g}^1$ mode remains insensitive to the doping level of MoS$_{2}$ crystals. Furthermore, recent findings from experimental and theoretical studies on the doping-dependent Raman spectroscopy of twisted bilayer MoS$_2$ samples reveal twist-angle-dependent softening and broadening of the A$_{1g}$ mode with electron doping, while the E$_{2g}^1$ mode remains mostly unaffected^42^. The A$_{1g}$ has a large electron-phonon coupling, and any variation in the electron density can strongly affect the coupling strength and hence the behaviour of the A$_{1g}$ mode. The linewidth (FWHM) of the A$_{1g}$ mode directly scales with the strength of electron–phonon coupling. In contrast, the coupling of E$_{2g}^1$ phonon mode with electrons is weakly dependent on doping.

Thus our experimental observations with the existing understanding, allows us to infer the following: (I) Since A$_{1g}$ phonon mode is insensitive to in-plane uniform strain, the anomalous reduction in FWHM of A$_{1g}$ mode observed in temperature-dependent Raman measurements is not a result of thermally induced strain (II) Since the anomalous response is only observed in the A$_{1g}$ mode it could be due to temperature-induced charge transfer/p-type doping developed in the sample (III) the absence of anomalous behavior in excitation power-dependent Raman measurements can be attributed to the interplay of photo-induced electron transfer from SiO$_2$ substrate to MoS$_2$ sample^35^, local temperature rise and laser-induced strain which overrides the effect of temperature induced charge transfer/p-type doping.

Previous reports showed that the mechanically exfoliated MoS$_{2}$ transferred to Si/SiO$_2$ substrate could have excess electrons due to its interaction with the substrate^14^. In addition, the presence of defects in the crystals could strongly influence the charge and phonon dynamics of the system. Here the X-ray photoelectron spectroscopy (XPS) measurements give the stoichiometry of our annealed MoS$_{2}$ crystal as Mo:S ratio in the range of 1:1.71 to 1:1.76, corresponding to 12–15% sulfur vacancies; see Section S6 in the supporting information for details. The existence of intrinsic sulfur vacancies is previously reported in mechanically exfoliated^43^ and CVD grown^44^ MoS$_{2}$ and other TMDCs with similar structures^45^. Hence, our molybdenum disulfide samples are natively n-type due to the electron-donating nature of sulfur vacancies in MoS$_{2}$^46^ and the SiO$_2$ layer in the substrate.

### Temperature dependent Photoluminescence measurements

**Figure 7:**
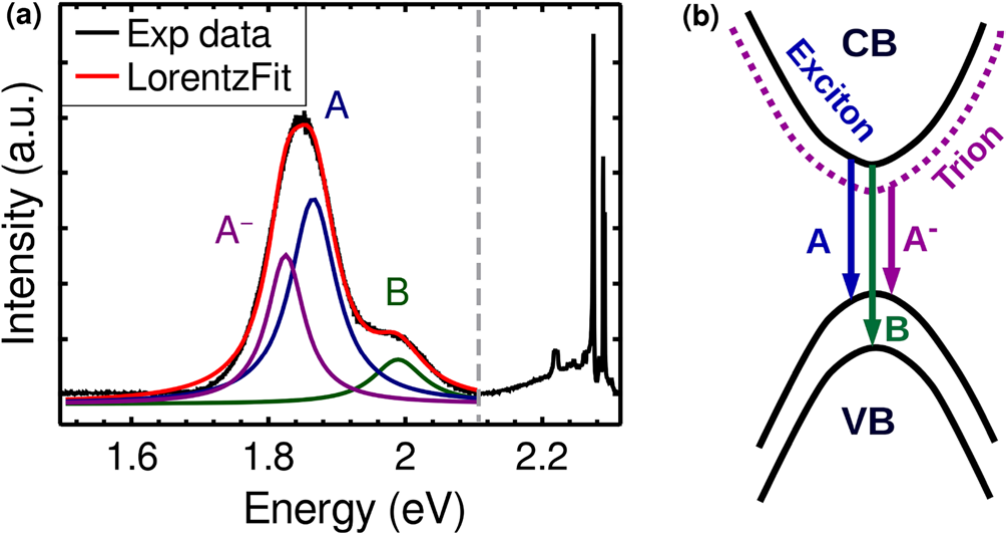
(**a**) The room temperature photoluminescence spectrum of monolayer MoS$_2$ on Si/SiO$_2$ substrate. The three peak fit corresponds to A and B excitons and A$^-$ trion. The peaks in the high energy are the Raman active phonon modes of MoS$_2$ and Si/SiO$_2$ substrate. (**b**) The schematic of the exciton and trion transitions.

To gain further insight into the charge carrier dynamics, photoluminescence measurements were carried out with 532 nm off-resonance excitation. Figure 7a shows the PL spectrum of supported monolayer MoS$_{2}$ at room temperature. Essentially the spectrum consists of a broad asymmetric peak centred at $\sim 1.85$ eV and a weak shoulder at $\sim 2$ eV. The spectrum is fitted with three Lorentzian peaks where the two excitonic peaks named A and B correspond to the direct transition. Besides excitons (electron–hole pairs), trions (also known as charged excitons) can also exist in 2D MoS$_{2}$ samples which are not observed in their bulk form. The peak labelled A$^-$ refers to the negatively charged trion created by an electron’s binding to an A exciton. It is important to note that only negatively charged trions are observed in MoS$_2$ which results from high electron density in the sample due to unintentional n-doping^6,47^. The band diagram in Fig. 7 (b) is a schematic of exciton and trion transitions. PL in MoS$_{2}$ has a strong dependence on layer number^10,15,48^, stacking order^48^, temperature^15,49^, strain^16^ and carrier density^17^. The PL spectrum corresponds to different layer numbers are provided in supporting Figure S10. As the layer number increases, the PL intensity corresponding to direct transition decreases significantly, as reported previously^10,15,48^.

**Figure 8:**
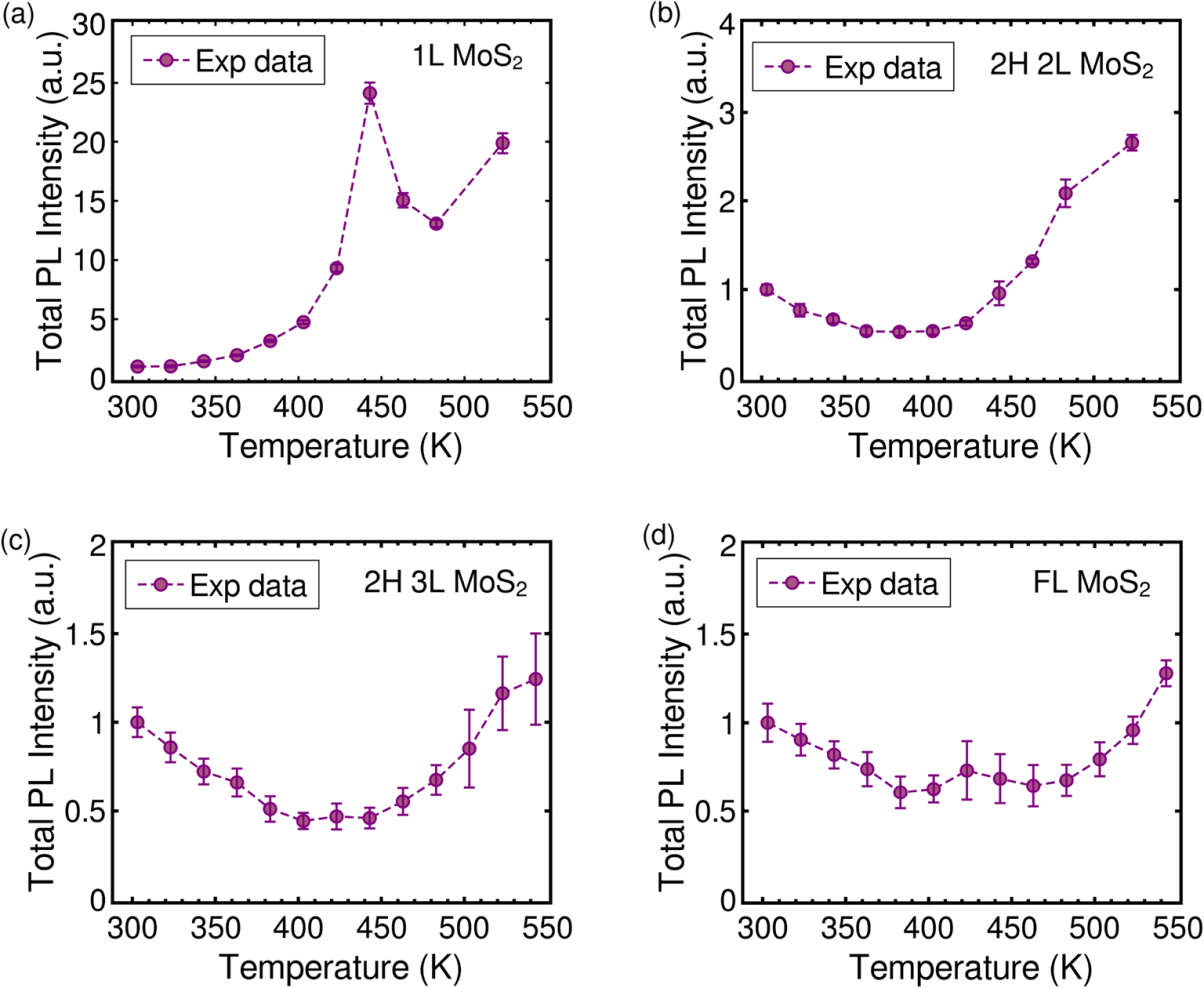
The variation of total integrated intensity of A and B excitons and A$^-$ trion emission (normalized by the spectral integrated intensity at 303 K) as a function of temperature for (**a**) mono-, (**b**) bi-, (**c**) tri-, and (**d**) few-layer MoS$_2$ flakes on Si/SiO$_2$. The dashed line is provided for eye-guide.

To investigate the temperature-induced carrier dynamics in the MoS$_2$ samples, temperature-dependent PL measurements are conducted. The variations of the PL spectrum with temperature are analyzed in both air and N$_2$ environments, and the results are provided in Section S8. As the temperature increases, the PL peaks of all our samples exhibit a redshift in energy, as depicted in Figure S14. This redshift is due to the bandgap narrowing arising from the increased electron-phonon interactions and small changes in the bonding length at higher temperatures^15,50–52^. The total PL intensity of the direct transition is generally expected to decrease with increasing temperature due to thermally activated phonons, which leads to an increase in nonradiative recombination owing to enhanced photocarrier-phonon interactions^15^. However, in this study, we observed an intriguing trend in the total PL intensity of MoS$_2$ samples. In the case of monolayer samples, the intensity initially increases as the temperature rises, followed by a sudden decrease after reaching 443 K, and then a subsequent increase at higher temperatures. This behavior is consistent in both air and N$_2$ environments, as illustrated in Fig. 8a and detailed in Section S8. On the other hand, for bilayer to few-layer samples, the PL intensity initially decreases with increasing temperature and begins to increase at higher temperatures. Notably, the temperature at which the PL intensity starts to increase depends on the layer number, as shown in Fig. 8b–d. Specifically, these temperatures are approximately 423 K, 463 K, and 503 K for bilayer, trilayer, and few-layer samples, respectively.

To understand the observed behaviour, we first focus on the temperature-dependent PL response of the monolayer sample. As the temperature increases, the intensity of A exciton dominates over the intensity of negative trion, see Figure S15. This can be attributed to the depletion of electrons due to the interaction of sulfur vacancies with air or N$_2$ at higher temperatures^12,53,54^. Prior studies have demonstrated that the PL intensity of monolayer MoS$_2$ can be modulated by p-type doping, resulting in an increase, or by n-type doping, leading to a decrease in PL intensity. This modulation in PL intensity also directly relates to the dominant PL mechanism (exciton or trion)^17^. Hence, the observed increase in total PL intensity in the monolayer sample could be attributed to a reduction in electron density at higher temperatures, thereby inducing temperature-induced p-type doping. However, this model does not explain the increases in PL intensity observed in the multilayer samples, nor their dependence on the layer number at higher temperatures.

The initial decrease in PL intensity observed in the multilayer samples with increasing temperature can be attributed to thermally-induced nonradiative recombinations within the system. The phenomenon of high-temperature PL enhancement for direct transitions in few-layer MoS$_2$ and other TMDCs has been previously reported and explained independently through the inter-layer decoupling and intervalley charge transfer mechanisms^50,55^. Interlayer decoupling occurs as a result of thermal expansion-induced separation between neighboring layers, leading to an indirect-to-direct bandgap transition at elevated temperatures^50^. However, given the low thermal expansion coefficient of MoS$_2$, achieving interlayer decoupling requires very high temperatures, making it unlikely to be the cause of the observed PL enhancements below 550 K. Li et al. proposed that the luminescence enhancement observed in few-layer MoS$_2$ above 520 K is a result of phonon-mediated intervalley charge transfer, where thermally activated carriers transfer from the $\Delta$/$\Gamma$ point to the K point of the Brillouin zone^55^. Our experimental findings with few-layer MoS$_2$ align with this intervalley charge transfer model. However, supporting literature for bi- and tri-layer samples is still lacking.

The indirect bandgap of MoS$_{2}$ decreases as the layer number increases^10^, whereas the direct bandgap remains less altered. Hence the thermally induced carrier generation and their transfer from $\Delta$/$\Gamma$ point to K point in bi or tri-layers may emerge/dominate at a lower temperature compared to few-layer number flakes. Additionally, there might be variations in the band structure of MoS$_2$ due to the presence of sulfur vacancies. These variations in the band structure could be the reason for the layer number dependence of high-temperature PL enhancement.

Next, we examine the consistency of our results from Raman and PL measurements. The observed reduction in FWHM of the A$_{1g}$ mode and the decrease in the relative intensity of the E$^1_{2g}$ mode to the A$_{1g}$ mode in the temperature-dependent Raman measurements of MoS$_2$ samples can be attributed to a decrease in electron density as the temperature increases. This decrease in electron density is a result of interactions between sulfur vacancies and O$_2$ or N$_2$ molecules in the environment at higher temperatures, primarily occurring at the exposed edges and top surface of the MoS$_2$ samples^12,53,54^. Consequently, the effects are more pronounced in monolayer samples and diminish as the layer number increases to a few layers. Furthermore, the observed hysteresis in the FWHM of the A$_{1g}$ mode and the relative intensity of the E$^1_{2g}$ mode to the A$_{1g}$ mode during the heating and cooling cycles confirms that these anomalous responses originate from the interactions occurring at the surfaces of the MoS$_2$ crystals at elevated temperatures. Additionally, our comparison of samples before and after annealing reveals that the anomalous responses begin to dominate at higher temperatures for the non-annealed samples; as discussed in Section S3. The annealing process creates more sulfur vacancies^12,56^, resulting in the onset of anomalous responses even at temperatures closer to room temperature. These observations further support the conclusion that the anomalous responses are attributed to the presence of sulfur vacancies in the crystals and their interactions with the environment at elevated temperatures.

In summary, this study presents a unique off-resonant temperature-dependent Raman and PL investigation of MoS$_2$ with inherent sulfur vacancies. The anomalous behavior of the A$_{1g}$ mode in the temperature-dependent Raman spectrum can be attributed to the reduction in electron density at higher temperatures. For monolayer samples, the high-temperature PL response is primarily influenced by the interaction of sulfur vacancies with molecules in the surrounding environment, while for multilayer samples, it is dominated by intervalley charge transfer. These findings unequivocally demonstrate that the high-temperature Raman and PL responses of 2D MoS$_2$ crystals are significantly influenced by the presence of defects (sulfur vacancies) within the crystal structure. Notably, the impact of vacancies is more pronounced in the intensity/relative intensity and FWHM of the peaks rather than in the peak position.

## Conclusions

We performed a detailed study of the temperature-dependent Raman and photoluminescence spectrum of MoS$_2$ by accounting for the effects of layer number and stacking order of the crystal. Followed by a detailed analysis of experimental observations, the temperature-induced anomalous Raman response of the A$_{1g}$ phonon mode and its layer number dependence is attributed to the variation in the electron density of the crystal with temperature. The unintentional sulfur vacancies are found to have a vital role where their interaction with air or N$_2$ environment at higher temperatures reduced the electron density of the sample, hence the electron–phonon interaction strength. The significant enhancement in the PL intensity of our monolayer samples also supports this mechanism. The layer number-dependent PL enhancement at high temperatures is correlated with the temperature-enhanced phonon mediated intervalley transfer of the carriers from $\Delta$/$\Gamma$ point to the K point of the Brillouin zone. We show that the effect of defects (sulfur vacancies) at high temperatures is reflected more in the FWHM and relative intensity of the Raman modes than in the Raman peak positions. The experimental correlations derived from our study, relating the relative intensity, full-width at half-maximum (FWHM), and temperature, provide valuable insights into the intricate defect dynamics exhibited by MoS$_2$ under elevated temperature conditions. These findings contribute significantly to the comprehension of defect behaviour and aid in advancing our understanding of MoS$_2$’s response to thermal variations. This enhanced understanding holds immense potential for defect engineering strategies aimed at diverse applications.

### Supplementary Information

Supplementary Information.

## Data Availability

All data that support the findings of this study are included in this published article and its Supplementary Information file. Raw data files if required are available from the corresponding authors on reasonable request.
