# Supplementary material for: High temperature anomalous Raman and photoluminescence response of molybdenum disulfide with sulfur vacancies
Source: Sci Rep. 2023 Sep 29;13:16418. doi: 10.1038/s41598-023-43756-w (PMC10541451; doi:10.1038/s41598-023-43756-w)
Supplement: Supplementary file 1 — Supplementary Information. [file 41598_2023_43756_MOESM1_ESM.pdf]

Supporting Information

High temperature anomalous Raman and photoluminescence response of molybdenum disulfide with sulfur vacancies

Ranjuna M K and Jayakumar Balakrishnan

Department of Physics, Indian Institute of Technology Palakkad, Palakkad-678623, Kerala, India

S1. Layer number and stacking order determination

S1. 1. Optical identification of MoS_2 layer number

Similar to other 2D materials, when MoS_2 is transferred onto a SiO_2/Si substrate and observed under an optical microscope, the intensity of reflected light from the 2D material differs from that of the bare substrate. For white light illumination and an oxide thickness of approximately 285 nm, the optical contrast of MoS_2 increases with the layer number[1]. Thus, the variation in optical contrast serves as a primary indicator of the layer number. Figure S1 presents some of the captured images.

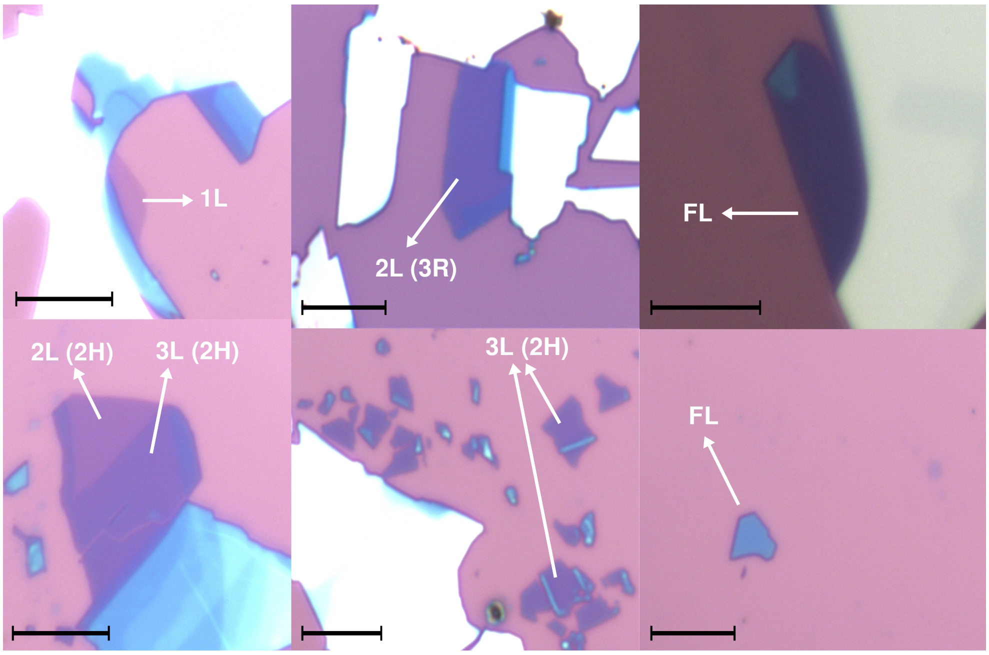

Figure S1: The representative optical images of some MoS_2 flakes on the SiO_2/Si substrate used in our study. The scale bar given in all images is equivalent to 10 μm. FL stands for few-layer.

S1. 2. Crystal structure and Stacking order

The most stable structure of monolayer MoS_2 consists of Mo centers coordinated with six sulfur ligands in a trigonal prismatic (1H) arrangement, lacking inversion symmetry. In the case of few-layer MoS_2, despite the identical crystal structure of each layer, stacking arrangements of 1H-MoS_2

layers give rise to two polytypes: hexagonal (2H) and rhombohedral (3R). Even though 2H is more stable than 3R, the coexistence of these two stacking types occurs in natural bulk crystals due to the similarity in their formation energies.[2, 3] As the layer number increases, the stacking order can vary significantly. For instance, trilayer MoS_2 can exhibit 2H, 3R, and mixed stacking orders.[4, 2] Prior studies have reported the presence of 2H, 3R, and mixed stacking sequences in few-layer MoS_2 obtained via mechanical exfoliation from natural molybdenite crystals [3], as well as commercially available single crystals [2].

The most reliable method to determine the stacking order in MoS_2 is through low-frequency Raman modes, specifically the layer breathing modes. In 3R-stacked MoS_2, the difference between the frequencies of the layer breathing mode and the shear mode is approximately 3 cm^{-1} less than that in 2H-stacked MoS_2. Notably, 2H MoS_2 shows an intense Raman signal in the highest-frequency shear mode, while 3R MoS_2 exhibits the strongest Raman response in the lowest-frequency shear mode[4]. The integrated intensity ratio of the layer breathing mode to the shear mode can also be used to distinguish between 2H and 3R stacking in bilayer MoS_2[5]. For bilayer MoS_2, the I(LB)/I(S) ratio for 2H stacking is close to 1, whereas for 3R stacking, it is higher than 5. The peak fit parameters used to distinguish the stacking order of bilayers in Figure S1 are summarized in Table S1. The low-frequency Raman spectra of the few-layer samples displayed in Figure S1 are given in Figure S2. In the spectrum acquisition process, we used linearly polarized incident excitation and detector with no specific polarization configuration.

Table S1: Summary of low-frequency Raman spectrum parameters of bilayer MoS_2

**Sample**	**Pos(LBM) in cm^{-1}**	**Pos(SM) in cm^{-1}**	**I(LB)/I(S)**
2H 2L MoS_2	41.18 ± 0.57	22.573 ± 0.050	1.31 ± 0.46
3R 2L MoS_2	39.02 ± 0.18	21.856 ± 0.091	6.2 ± 1.1

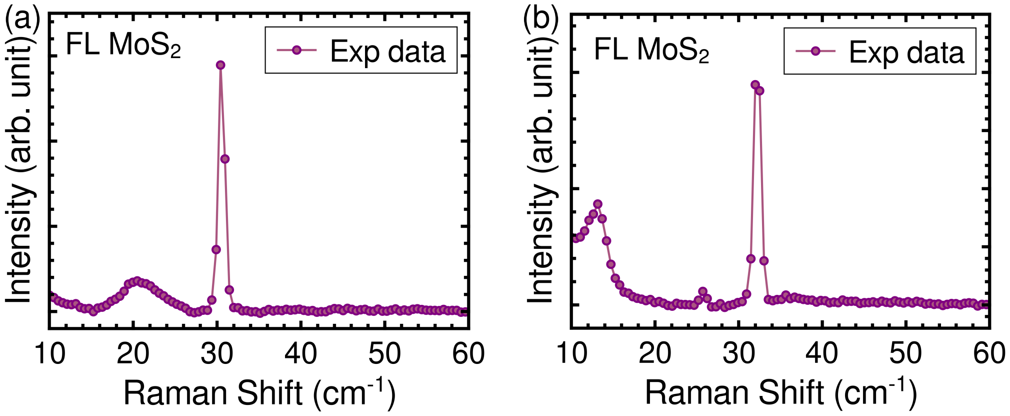

Figure S2: Low-frequency Raman spectra of few-layer MoS_2 flakes identified as (a) dark blue and (b) blue-green in the corresponding optical images shown in Figure S1, recorded at room temperature.

S2. Temperature-dependent Raman Spectra

The Raman spectra of mono-, bi-, tri-, and few-layer MoS_2 samples recorded at different temperatures are presented in Figure S3. To facilitate a detailed analysis of the observed trends, the variation of spectral parameters such as peak position, Full Width at Half Maximum (FWHM), relative intensity, and integrated intensity with increasing temperature is plotted in Figure 2 and Figure 3 of the manuscript. The anomalous responses are particularly evident in the mono-, bi-, and trilayer samples

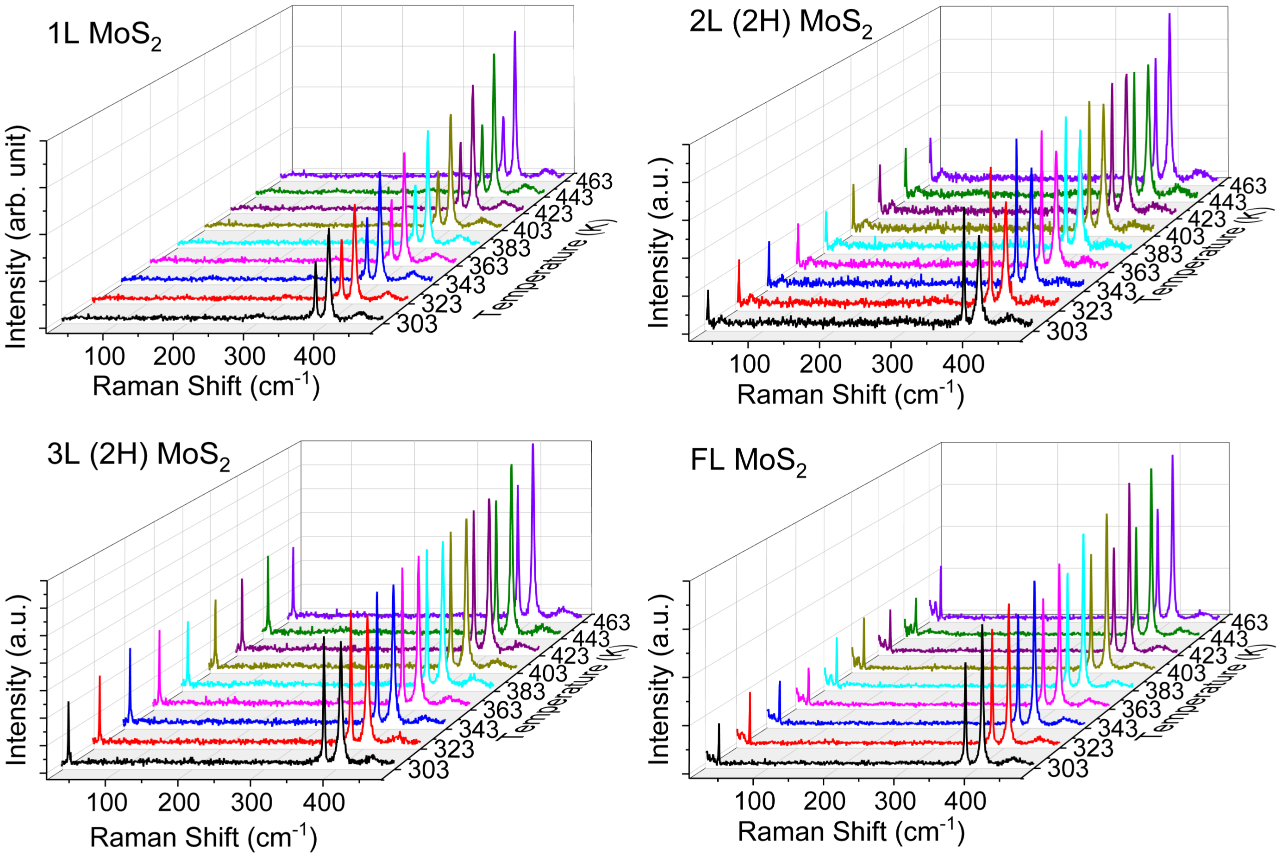

Figure S3: Raman spectrum of mono-, bi-, tri- and few-layer MoS_2 flakes recorded at different temperatures. All flakes are exfoliated on Si/SiO_2 substrate.

Estimated first-order temperature coefficients of E^1_{2g} and A_{1g} modes are compared with available literature and summarized in Table S2.

Table S2: Summary of first-order temperature coefficients of E^1_{2g} and A_{1g} modes of MoS_2

**Sample details**	**First-order temperature coefficient ($cm^{-1}K^{-1}$)** $\mathbf{E^1_{2g}}$	$\mathbf{A_{1g}}$	**Reference**
1L MoS_2 on SiO_2/Si	-0.0168 ± 0.0005	-0.015 ± 0.001	This work
2L MoS_2 (2H) on SiO_2/Si	-0.0161 ± 0.0004	-0.0113 ± 0.0006	
2L MoS_2 (3R) on SiO_2/Si	-0.0164 ± 0.0002	-0.0119 ± 0.0009	
3L MoS_2 (2H) on SiO_2/Si	-0.0148 ± 0.0005	-0.0126 ± 0.0004	
FL MoS_2 on SiO_2/Si	-0.0142 ± 0.0004	-0.0127 ± 0.0003	
2L MoS_2 (2H) on SiO_2/Si	-0.016 ± 0.001	-0.011 ± 0.003	Ref [5]
2L MoS_2 (3R) on SiO_2/Si	-0.017 ± 0.001	-0.013 ± 0.002	
1L MoS_2 on SiO_2/Si	-0.013	- 0.012	Ref [6]
2L MoS_2 on SiO_2/Si	-0.013	- 0.012	
5L MoS_2 on SiO_2/Si	-0.0128	- 0.0124	Ref [7]
1L MoS_2 on Au/SiO_2/Si		-0.0167 ± 0.0007	Ref [8]
2L MoS_2 on Au/SiO_2/Si		-0.0139 ± 0.0003	
1L MoS_2 suspended		-0.0203 ± 0.0006	
2L MoS_2 suspended		-0.0136 ± 0.0006	
1L MoS_2 suspended	-0.011 ± 0.001	-0.013 ± 0.001	Ref [9]
1L MoS_2 on Sapphire	-0.017	-0.013	
FL MoS_2 suspended	-0.0132	–0.0123	Ref [10]
Bulk MoS_2	-0.0147	-0.0123	Ref [11]

S3. Temperature-dependent Raman measurements before annealing

The temperature-dependent Raman measurements were performed on exfoliated MoS_2 samples both before and after annealing in an Ar-H environment. The annealing of MoS_2 in an Ar-H_2 environment for more than 300°C can create additional sulfur vacancies in the sample[12]. The measurements were repeated on multiple samples with the same layer number to account for any sample-to-sample variations. The temperature dependence of Raman spectral parameters for some of the exfoliated mono- and bilayer samples before annealing is shown in Figure S4. It is observed that the reduction in the FWHM of the A_{1g} mode occurs at higher temperatures compared to the annealed samples. Additionally, there is some sample-to-sample variation, which can be attributed to slight differences in the sulfur vacancy concentration within the samples. The corresponding data for some of the exfoliated few-layer samples before annealing is presented in Figure S5. Notably, an increase in the FWHM of the A_{1g} mode is clearly observed in few-layer MoS_2 samples with greater thicknesses, confirming the absence of anomalous Raman behavior in these few-layer samples. Therefore, even when considering sample-to-sample variations for the same layer numbers, it is evident that the anomalous responses diminish as the layer number increases and disappear as the layer number reaches a few layers.

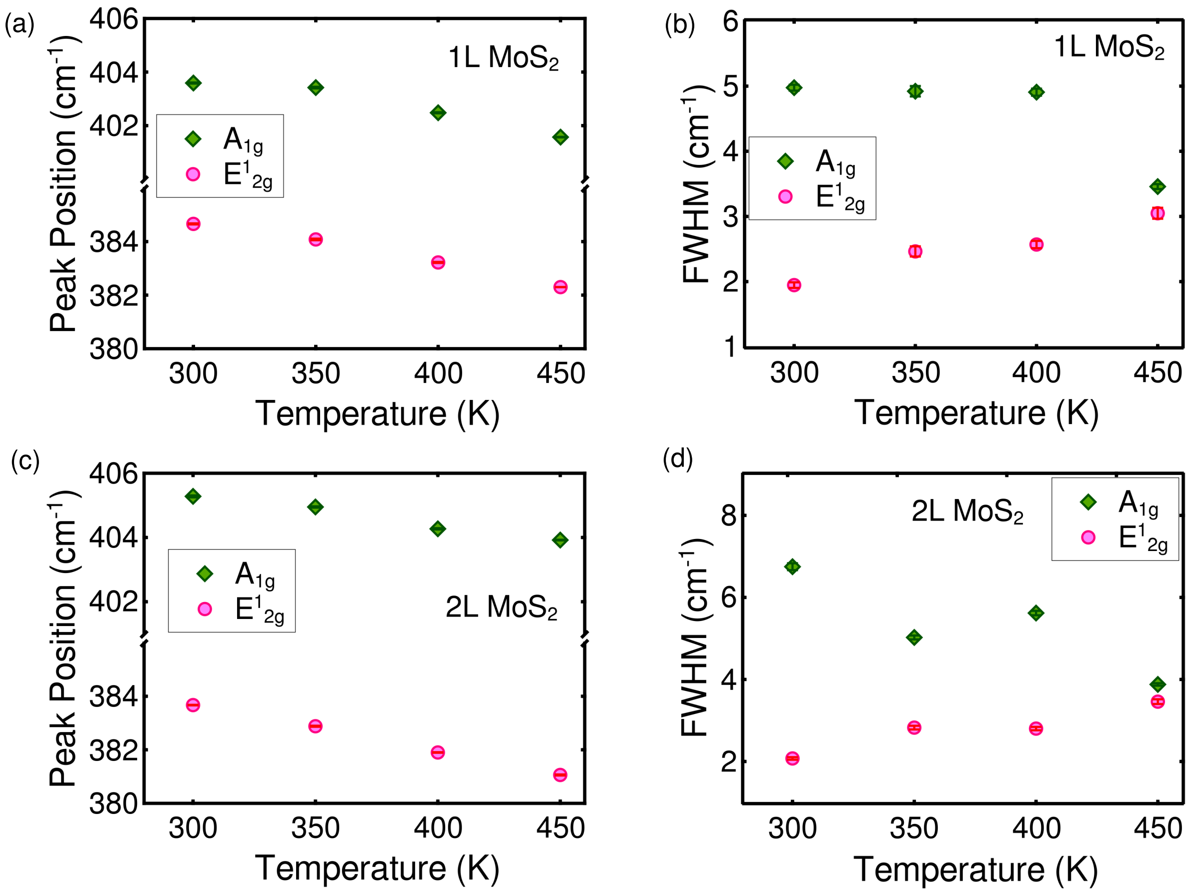

Figure S4: Variation of phonon frequency (left panel) and FWHM (right panel) of (a-b) mono- and (c-d) bilayer MoS_2 samples before annealing.

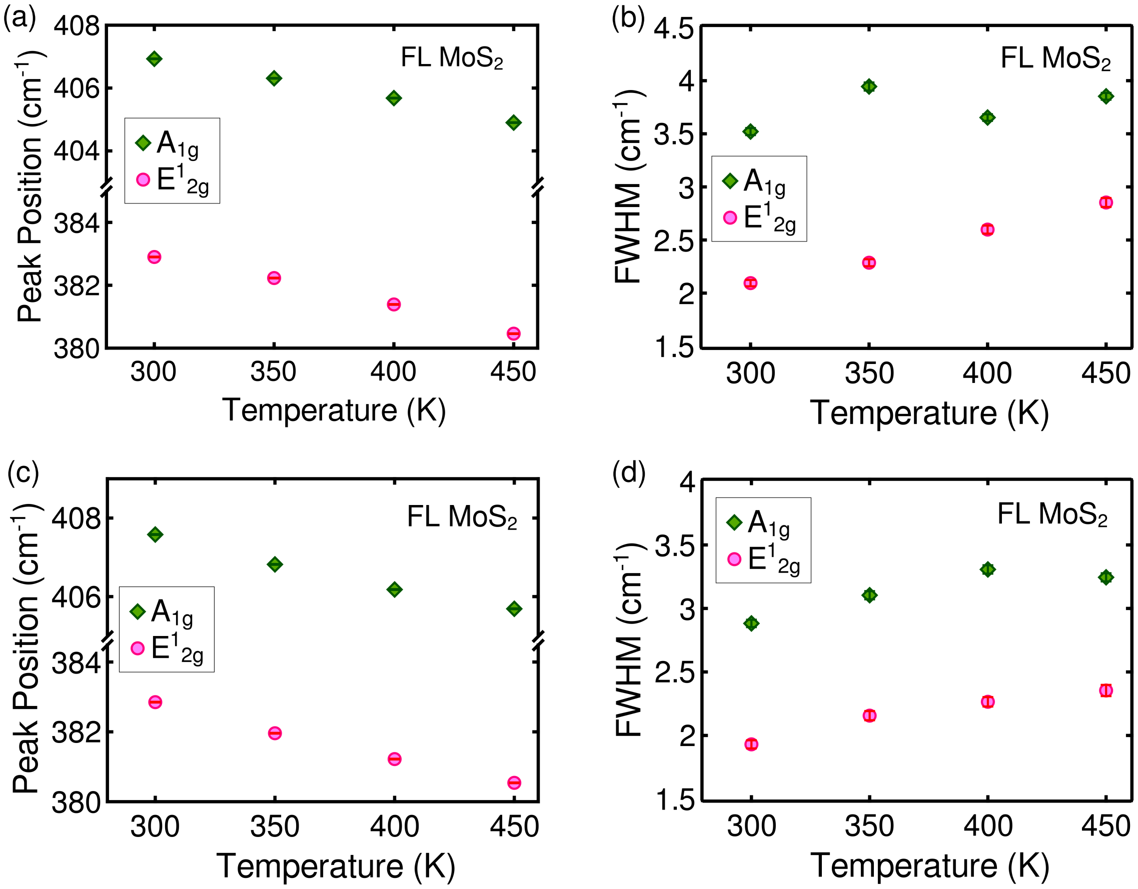

Figure S5: Variation of phonon frequency (left panel) and FWHM (right panel) of (a-b) few-layer MoS_2 samples before annealing (from top to bottom thickness increasing).

S4. Temperature-dependent Raman measurements of 3R stacked bilayer

The temperature-dependent Raman measurement results of 3R stacked bilayer MoS_2 samples are presented in Figure S6. Similar to 2H stacked bilayers, the anomalous behavior of the FWHM of the A_{1g} mode and the relative intensity of the A_{1g} mode to the E^1_{2g} mode is observed in 3R stacked bilayer samples. Thus, the anomalous response is not specific to only one type of stacking order.

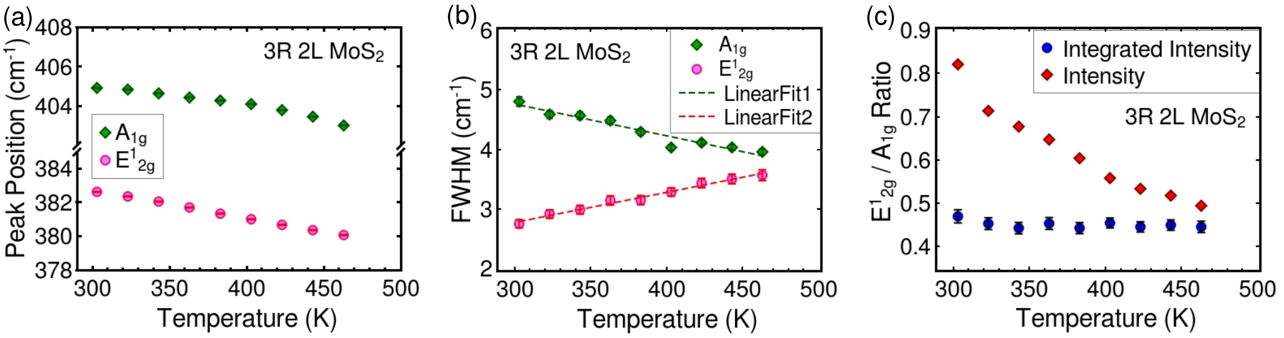

Figure S6: Variation of (a) peak position, (b) FWHM and (c) intensity ratio and integrated intensity ratio of high-frequency Raman modes of 3R stacked bilayer MoS_2

S5. Laser power-dependent Raman measurements

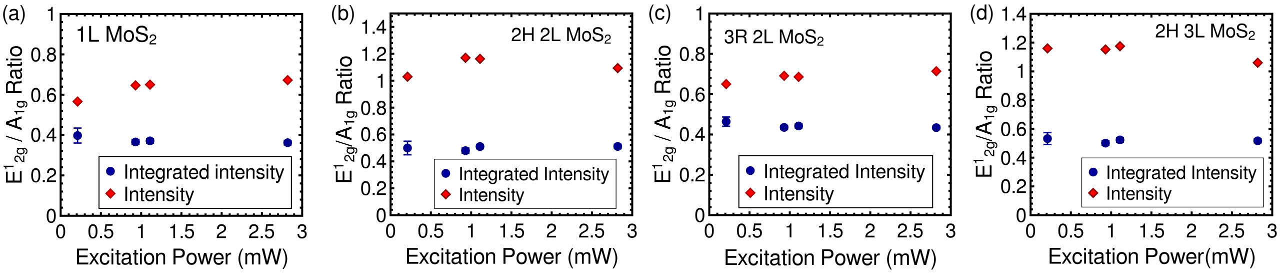

Figure S7: Variation of E^1_{2g} to A_{1g} integrated intensity ratio and intensity ratio for (a) mono-, (b) 2H stacked bi-, (c) 3R stacked bi- and (d) 2H stacked tri-layer MoS_2

S6. Stoichiometry determination

Scanning electron microscopy (SEM) and Energy-dispersive X-ray spectroscopy (EDS) used for the primary measurement of the composition and atomic percentage of various elements in our sample. EDS mapping was performed on exfoliated few-layer MoS_2 flakes at different locations of the substrate. The EDS spectrum, shown in Figure S8, displays characteristic X-ray energies in keV on the X-axis and intensity in counts on the Y-axis. Peaks corresponding to silicon, oxygen, molybdenum, and sulfur were observed. The peak intensities are proportional to the element concentrations. The atomic percentage of Mo and S obtained for different flakes ranged from 1:1.70 to 1:1.93. The lowest sulfur content was observed when scanning over the areas covering the edges of the top layers in continuous few-layer regions.

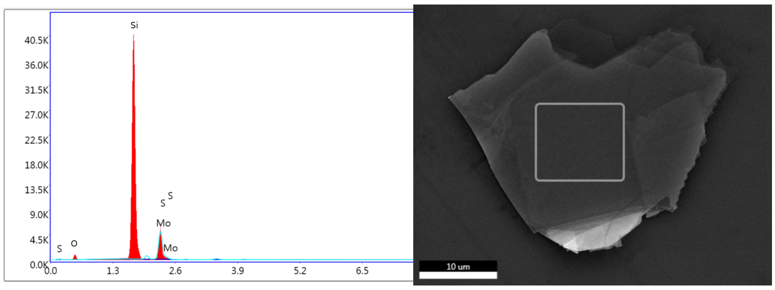

Figure S8: EDS spectrum of MoS_2 flake on Si/SiO_2 (left) and the SEM image of the flake (right). The EDS mapping area is highlighted in white colour box

X-ray Photoelectron Spectroscopy (XPS) measurements were conducted using non-monochromatic Mg Kα radiation (1253.6 eV). As the XPS probe size is approximately 6 mm, both the exfoliated MoS_2 flakes and the bare substrate regions were exposed to the probe. Consequently, in the survey scan, peaks corresponding to Molybdenum, Sulfur, Silicon, Oxygen core levels, and a carbon 1s peak were obtained. The C 1s peak was used for charge correction. The Mo 3d core level peaks were fitted with two doublets corresponding to Mo(+6) and Mo(4+) oxidation states, while the S 2p peaks were fitted with one doublet, as shown in Figure S9. Position and area constraints were applied to each set of doublets. Finally, the stoichiometry of the annealed MoS2 crystal was determined to be in the range of 1:1.71 to 1:1.76, corresponding to 12-15% sulfur vacancies. It is worth noting that this value represent the upper limit of sulfur vacancy concentration since the area exposed to the XPS probe includes edge regions as well.

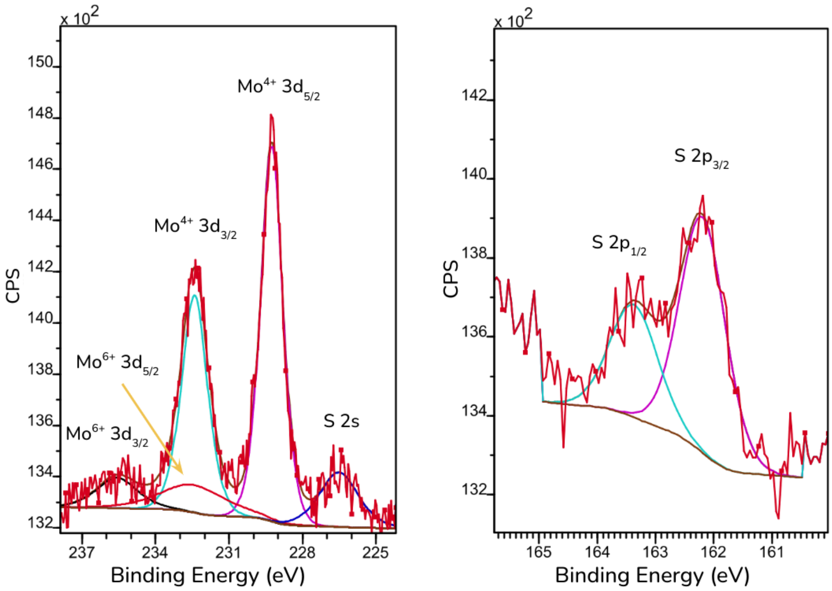

Figure S9: High resolution XPS spectrum of Mo 3d core and S 2p core. The baseline and the component fit are given in different colours.

S7. Layer-dependent PL measurements

As the layer number increases the PL intensity decreases.

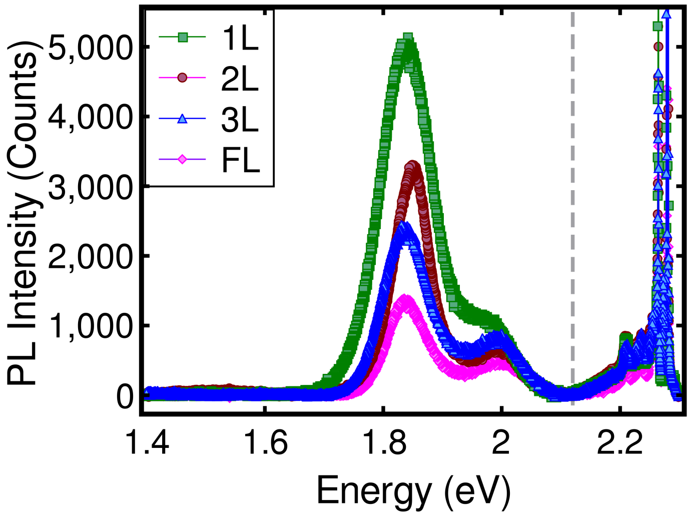

Figure S10: Photoluminescence spectrum of mono-, bi-, tri- and few-layer MoS_2 flakes on Si/SiO_2 substrate. The peaks at energy above 2.1 eV corresponds to Raman modes of MoS_2 and the substrate. The dotted line provided to distinguish PL and Raman peaks.

S8. Temperature-dependent PL measurements

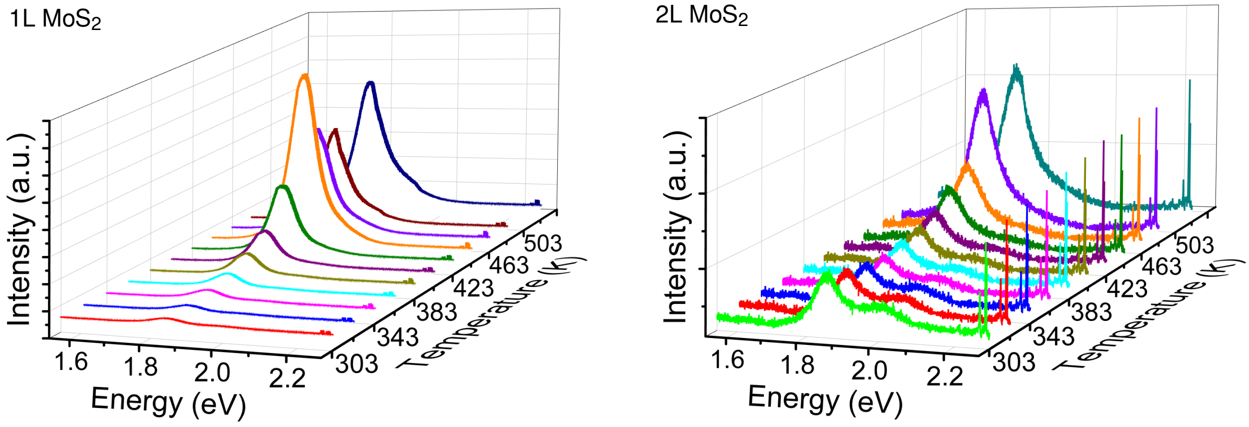

Figure S11: Total Photoluminescence spectrum recorded at different temperatures from mono- and bilayer MoS_2 flakes on Si/SiO_2 substrate in N_2 environment.

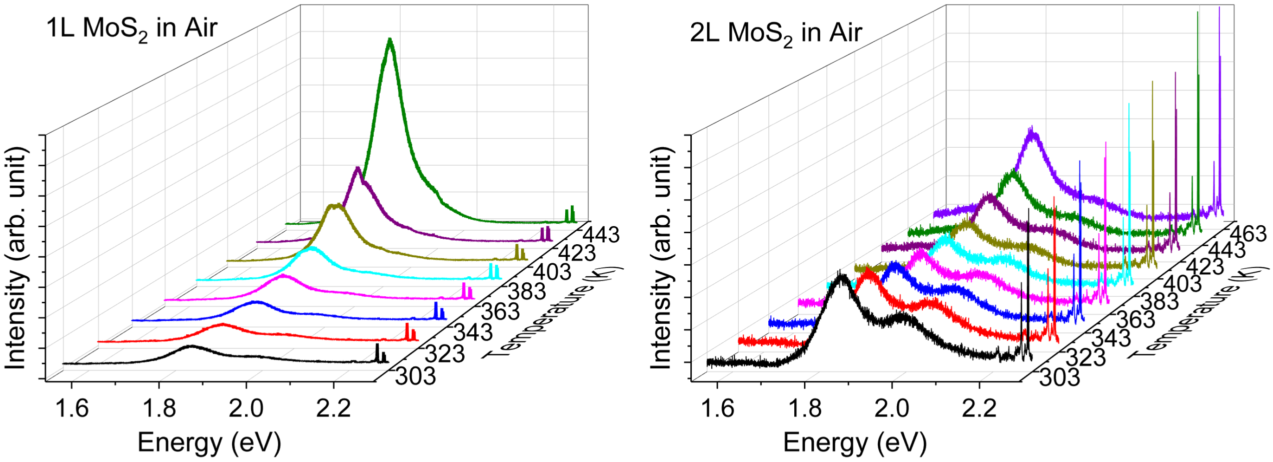

Figure S12: Total Photoluminescence spectrum recorded at different temperatures from mono- and bilayer MoS_2 flakes on Si/SiO_2 substrate in air.

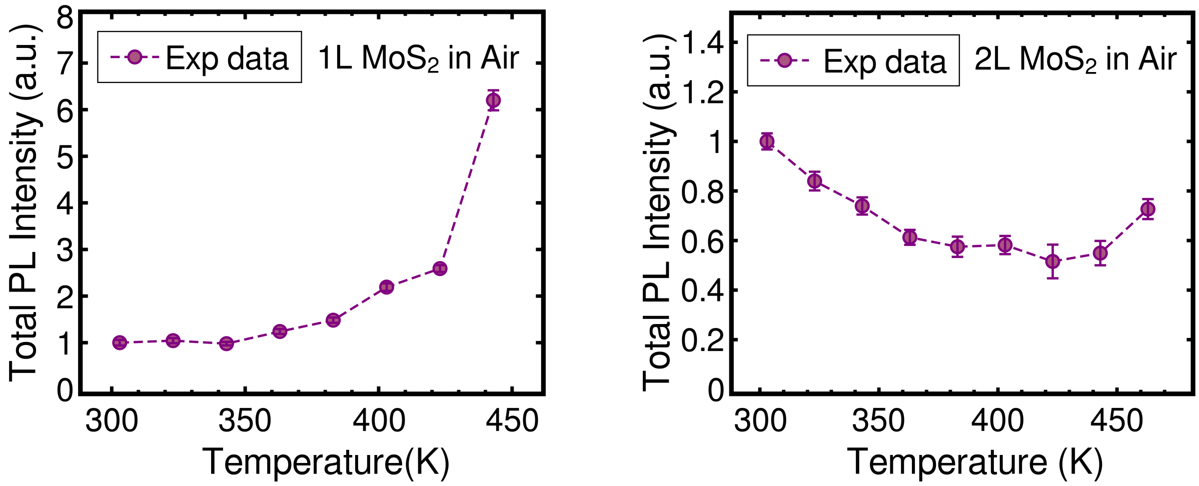

Figure S13: Total Photoluminescence integrated intensity (normalized by the integrated intensity at 303 K) of mono- and bilayer MoS_2 flakes on Si/SiO_2 substrate measured in air environment. The circles represent experiment data and the dotted line provided for eye guide.

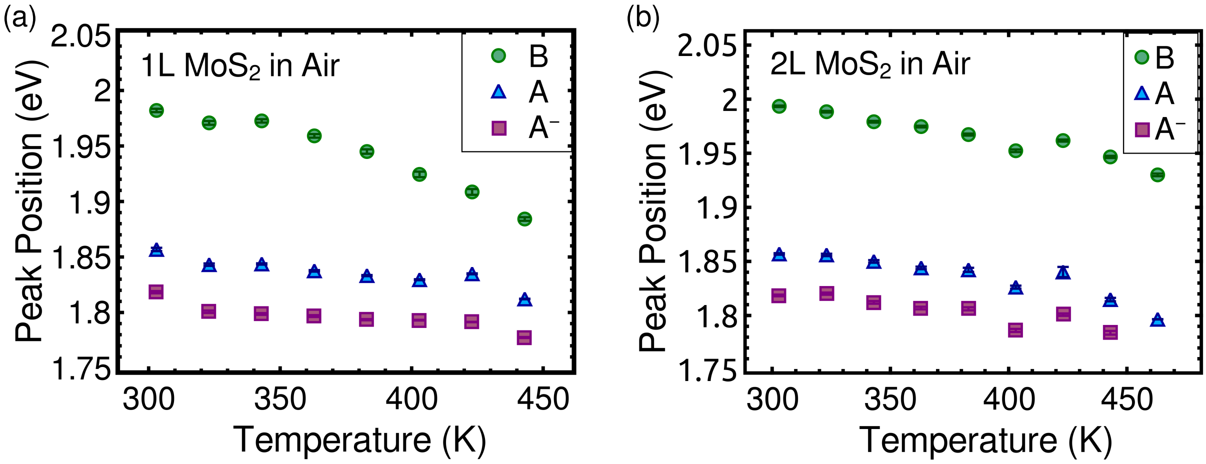

Figure S14: The variation of PL peak energy of B, A excitons and A^- trion as a function of temperature for (a) monolayer and (b) bilayer MoS_2 supported on Si/SiO_2 substrate and kept in air environment.

S8.1. Interaction of sulfur vacancies with environment

The interaction between N_2 or O_2 molecules in the environment and sulfur vacancies in the crystal is reversible in nature. Physisorption of N_2 or O_2 molecules onto sulfur vacancy sites involves charge transfer from MoS_2 to these molecules, leading to a reduction in the free carrier density of MoS_2.[13] Consequently, the intensity of the A^- trion decreases due to the reduced availability of a second electron for binding (since the trion is formed by the binding of a free electron to the A exciton).[13] As the temperature increases, more and more molecules in the environment interact with the MoS_2 surface, resulting in a continuous decrease in electron density. This, in turn, leads to a progressive decrease in A^- trion intensity; see Figure S15. Eventually, as the thermal energy surpasses the trion binding energy, the trion peak completely disappears.

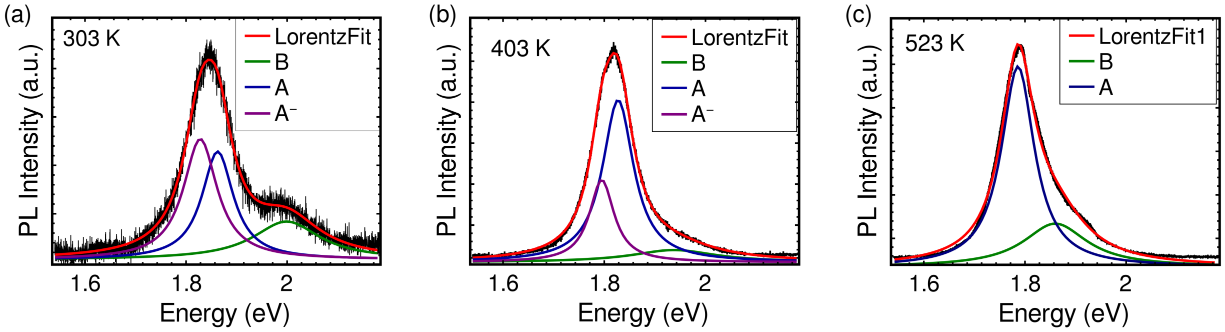

Figure S15: Photoluminescence spectrum of monolayer MoS_2 flakes on Si/SiO_2 substrate in N_2 at environment recorded at (a) 303 K, (b) 403 K and (c) 523 K. Peak fit corresponds to excitons and trion transitions.

References

[1] Nikhil Joseph Joy, MK Ranjuna, and Jayakumar Balakrishnan. A simple and robust machine learning assisted process flow for the layer number identification of tmds using optical contrast spectroscopy. *Journal of Physics: Condensed Matter*, 51(2):025901, 2022.

[2] Woongki Na, Kangwon Kim, Jae-Ung Lee, and Hyeonsik Cheong. Davydov splitting and polytypism in few-layer mos2. *2D Materials*, 6(1):015004, 2018.

[3] Jae-Ung Lee, Kangwon Kim, Songhee Han, Gyeong Hee Ryu, Zonghoon Lee, and Hyeonsik Cheong. Raman signatures of polytypism in molybdenum disulfide. *ACS nano*, 10(2):1948–1953, 2016.

[4] Jeremiah Van Baren, Gaihua Ye, Jia-An Yan, Zhipeng Ye, Pouyan Rezaie, Peng Yu, Zheng Liu, Rui He, and Chun Hung Lui. Stacking-dependent interlayer phonons in 3r and 2h mos2. *2D Materials*, 6(2):025022, 2019.

[5] Hanul Kim, Hayoung Ko, Soo Min Kim, and Heesuk Rho. Temperature dependent raman spectroscopy of shear and layer breathing modes in bilayer mos2. *Current Applied Physics*, 25:41–46, 2021.

[6] Suman Sarkar, Indrajit Maity, HL Pradeepa, Goutham Nayak, Laetitia Marty, Julien Renard, Johann Coraux, Nedjma Bendiab, Vincent Bouchiat, Sarthak Das, et al. Anharmonicity in raman-active phonon modes in atomically thin mos 2. *Physical Review B*, 101(20):205302, 2020.

[7] Xiao Fei Yue, Ying Ying Wang, Yi Zhao, Jie Jiang, Kai Yu, Yao Liang, Bo Zhong, Shou Tian Ren, Ren Xi Gao, and Ming Qiang Zou. Measurement of interfacial thermal conductance of few-layer mos2 supported on different substrates using raman spectroscopy. *Journal of Applied Physics*, 127(10):104301, 2020.

[8] Xian Zhang, Dezheng Sun, Yilei Li, Gwan-Hyoung Lee, Xu Cui, Daniel Chenet, Yumeng You, Tony F Heinz, and James C Hone. Measurement of lateral and interfacial thermal conductivity of single-and bilayer mos2 and mose2 using refined optothermal raman technique. *ACS applied materials & interfaces*, 7(46):25923–25929, 2015.

[9] Rusen Yan, Jeffrey R Simpson, Simone Bertolazzi, Jacopo Brivio, Michael Watson, Xufei Wu, Andras Kis, Tengfei Luo, Angela R Hight Walker, and Huili Grace Xing. Thermal conductivity of monolayer molybdenum disulfide obtained from temperature-dependent raman spectroscopy. *ACS nano*, 8(1):986–993, 2014.

[10] Satyaprakash Sahoo, Anand PS Gaur, Majid Ahmadi, Maxime J-F Guinel, and Ram S Katiyar. Temperature-dependent raman studies and thermal conductivity of few-layer mos2. *The Journal of Physical Chemistry C*, 117(17):9042–9047, 2013.

[11] Jl A Wilson and AD Yoffe. The transition metal dichalcogenides discussion and interpretation of the observed optical, electrical and structural properties. *Advances in Physics*, 18(73):193–335, 1969.

[12] Yiru Zhu, Juhwan Lim, Zhepeng Zhang, Yan Wang, Soumya Sarkar, Hugh Ramsden, Yang Li, Han Yan, Dibya Phuyal, Nicolas Gauriot, et al. Room-temperature photoluminescence mediated by sulfur vacancies in 2d molybdenum disulfide. *ACS nano*, 17(14):13545–13553, 2023.

[13] Sefaattin Tongay, Joonki Suh, Can Ataca, Wen Fan, Alexander Luce, Jeong Seuk Kang, Jonathan Liu, Changhyun Ko, Rajamani Raghunathanan, Jian Zhou, et al. Defects activated photoluminescence in two-dimensional semiconductors: interplay between bound, charged and free excitons. *Scientific reports*, 3(1):2657, 2013.
